# General Epidemiology of Animal Viruses

**DOI:** 10.3390/ani14213045

**Published:** 2024-10-22

**Authors:** Anna Szczerba-Turek

**Affiliations:** Department of Epizootiology, Faculty of Veterinary Medicine, University of Warmia and Mazury in Olsztyn, Oczapowskiego 13, 10-718 Olsztyn, Poland; a.szczerba@uwm.edu.pl

In recent years, the study of animal viruses and their epidemiology has been central to understanding the dynamics of viral infections in humans, livestock, wildlife, and pets [1,2,3]. The COVID-19 pandemic has demonstrated that these viruses can significantly affect not only animal health and agricultural productivity but also human health. Thanks to advancements in biotechnology, molecular biology, and virology, we now have powerful tools to study viral variants and their determinants, distribution, and prevalence. These viruses spread through various routes, including direct contact, aerosol transmission, vector-borne transmission, and environmental persistence. Understanding these transmission mechanisms is crucial for developing effective control strategies and preventive measures [1,2,3].

Another significant challenge associated with animal viruses is the impact of globalization and climate change. Increased trade, animal movement, and shifting environmental conditions have heightened the risk of emerging viral diseases. Continuous monitoring of animal virus epidemiology is therefore essential to safeguard both human and animal health. Surveillance systems, when integrated with molecular techniques, enhance our ability to detect and respond to outbreaks, reducing the risks these viruses pose [4,5].

This comprehensive exploration of animal virus epidemiology covers key areas, including virus classification, transmission dynamics, epidemic investigation methodologies, and the role of vaccination and biosecurity in disease control. By synthesizing current knowledge and research, we aim to provide a deeper understanding of how animal viruses operate within populations and their implications for public health, agriculture, and wildlife conservation [6,7,8,9,10].

I am pleased to present this Special Issue, entitled the “General Epidemiology of Animal Viruses”, which features a collection of eleven articles offering significant insights into the world of animal virology. This compilation is intended to provide a comprehensive overview of the latest research, with a focus on molecular epidemiology, virus–host interactions, virus evolution, and the implications for animal and public health.

Viruses have long been companions to both animals and humans, as highlighted by several studies within this Special Issue. The scope of this collection ranges from highly localized studies, such as the investigation of *Penguin Circovirus* in Namibia [11], to broader examinations, like the first serological detection of *Akabane Virus* in Egyptian cattle [12]. Both studies showcase the expanding boundaries of viral epidemiology, where advanced molecular techniques help identify and monitor viral presence even in seemingly distant environments.

The detection of *Bovine Group A Rotavirus* strains in Vietnam [13], alongside phylogenetic analyses of *Chaphamaparvovirus* in cats and dogs, further underscores the global diversity of viruses and their capacity for cross-species transmission [14]. These findings highlight the importance of understanding virus ecology, as they provide key insights into virus adaptation and potential zoonotic threats.

Notably, the epidemiology and control efforts of rabies in Brazilian cattle and equines provide a critical case study of disease management in rural ecosystems [15]. This work emphasizes the ongoing need for vigilant surveillance and the application of preventive measures, even in areas where disease trends appear to be on the decline. Meanwhile, in Italy, a retrospective study on infectious *Bovine Rhinotracheitis* eradication efforts offers valuable lessons for post-eradication disease monitoring, with a specific focus on cross-reactivity challenges [16].

In addition to these epidemiological perspectives, this Special Issue also sheds light on technological advancements in virus detection and storage, such as the use of FTA cards for the stabilization of RNA viruses, including *Avian influenza* and *Newcastle* disease [17]. These innovations, along with the genomic characterization of various viruses such as *Turkey Reovirus*, *Parrot Bornavirus*, *Aichivirus*, and *Aleutian Mink Disease Virus*, enrich our understanding of viral evolution and provide practical applications for disease control [18,19,20,21].

The studies featured in this Special Issue significantly advance our understanding of animal viruses, from molecular epidemiology and viral evolution to their broader impacts on animals and public health. The wide range of species and viruses studied highlights the need for a global, interdisciplinary approach to addressing viral threats. Ongoing research in this field is crucial for improving disease prevention, strengthening biosecurity, and safeguarding both animal and human populations from future outbreaks.

I extend my sincere appreciation to all the authors for their contributions, as well as the reviewers for their insightful evaluations and the editorial team for their support throughout this process. This collection represents a key milestone in the study of animal viruses and will hopefully serve as a valuable resource for researchers, veterinarians, and policymakers.

With the increasing recognition of the interconnectedness between animal and human health, I encourage continued collaboration and research in this field as we work toward a safer and healthier future for all species.

This Special Issue underscores the importance of collaboration in tackling the epidemiological challenges posed by animal viruses. The breadth of research, spanning from genomic analysis to field epidemiology, represents the diverse strategies needed to combat viral threats effectively. I would like to extend my sincere thanks to all the contributors for their valuable research and to the peer reviewers for their insights, which greatly enhanced the quality of this Special Issue.

I hope that this Special Issue will serve as a valuable resource for researchers, veterinarians, and public health professionals, contributing to the broader understanding and control of animal viruses. The insights garnered from these studies are crucial for advancing both basic and applied research in virology, epidemiology, and disease control.
